# Feasibility of using experimental high viscosity silicone oils: a pilot study

**DOI:** 10.1186/s40942-017-0105-8

**Published:** 2018-01-08

**Authors:** Shira Sheen-Ophir, Mordechai Rosner, Alexander Rubowitz

**Affiliations:** 1 Department of Ophthalmology, Meir Hospital and Sackler Faculty of Medicine, Tel Aviv University, 59 Tchernichovsky St., 44281 Kfar Saba, Israel; 2 Department of Ophthalmology, Sackler Faculty of Medicine, Tel Aviv University, Tel Aviv, Israel; 3 Goldschleger Eye Institute, Sheba Medical Center, Tel Hashomer, Israel

**Keywords:** Emulsification, Endotamponade, Proliferative vitreoretinopathy, Retinal detachment, Silicone oil

## Abstract

**Background:**

Polydimethyl silicones (silicone oils) are used in complex retinal surgeries, including difficult or recurrent retinal detachments, severe eye trauma, and other indications for long term tamponade. Two major problems with currently available silicone oils are emulsification and recurrent retinal detachment. The primary endpoint of this study was to evaluate the toxicity and safety of high viscosity silicone oils and second, the feasibility of using them with currently available vitrectomy systems.

**Methods:**

In this experimental study, 8 eyes of 8 New Zealand White rabbits underwent vitrectomy. The vitreous cavities of 2 eyes were filled with medical grade 5500 cS silicone oil, 4 eyes with 12,500 cS oil, and 2 eyes with 30,000 cS oil for 3 months, after which the animals were sacrificed and the eyes sent for histopathological examination. The duration required to inject 5 cc each of 1300, 12,500 and 30,000 cS oils, using a commercially available system (Alcon VFC Pac) were also evaluated.

**Results:**

Retinal histopathology was comparable in all eyes, with no excess toxic effect or damage seen in eyes with experimental oils. All oils were readily injectable with the VFC Pac system.

**Conclusions:**

High viscosity experimental silicone oils have similar chemical and physical properties to lower viscosity oils currently used. Therefore, toxicities are expected to be similar. In a small pilot sample of 8 rabbit eyes filled with silicone oil for 3 months, histopathology in eyes with 12,500, 30,000 cS or medical grade 5500 cS silicone oil were similar. Injecting these oils using available vitrectomy equipment is feasible. New, high viscosity silicone oils may offer several advantages over currently available oils for some patients.

## Background

Polydimethyl silicones (silicone oils) are used for long term endotamponade in complex retinal surgeries, including difficult or recurrent retinal detachments, severe eye trauma, and other indications [1, 2]. Proliferative vitreoretinopathy (PVR), the most common cause of surgical failure in retinal detachment surgery, occurs in approximately 5–10% of detachments, and is one of the main uses of long term endotamponade with silicone oil. Two major problems with currently available silicone oils are emulsification and recurrent retinal detachment [3, 4].

Emulsification, the formation of small oil droplets at the interface between oil bubbles and intraocular fluids or tissues, causes dispersion of these droplets into the aqueous and vitreous humors, with consequently higher risk of recurrent detachment, inflammation, secondary glaucoma, and keratopathy [5–7]. Emulsification occurs in 1% after 1 month, 11% at 3 months, 85% at 6 months, and 100% after 12 months [4, 5] and increases with decreasing oil viscosity. However, commercially available 1000 cS and 5000 cS silicone oils may not have clinically significant differences in emulsification [8].

Currently, there is no effective treatment for retinas that detach after placement of silicone oil and a large percentage of these eyes remain chronically detached [2, 4, 9–11]. This pilot study examined whether the use of high viscosity silicone oils is safe and feasible using currently available surgical equipment. We intended this as a first step toward examining the possibility of their applicability to provide improved tamponade in cases that failed with currently available silicone oils, and to decrease the incidence of emulsification.

As all dimethyl silicones are chemically identical, the different viscosities of different types of silicone oil arises from the differing lengths of the polymer chains. Increasing the molecular weight of the silicone oil results in a longer polymer chain and increased viscosity [7]. There is therefore, reason to think that high viscosity silicone oils will be as safe and as non-toxic as oils currently in medical use.

We hypothesized that high viscosity silicone oils, ranging from 12,500 to 30,000 centiStokes (cS), in contrast to currently used 1000–5500 cS oils, may provide long-term effective tamponade in patients who experience retinal detachment with currently used silicone oils, possibly with lower risk of long-term emulsification. Towards this end, this pilot study first examined whether silicone oils appear to be as safe and non-toxic to the retina as currently used lower viscosity silicone oils are and second, whether it is practical to inject high viscosity using current vitrectomy equipment.

## Methods: overview

A total of 8 eyes of 8 New Zealand White rabbits weighing 2–3 kg were included in the study. All experiments were approved by the local Animal Research Review Committee and performed in accordance with The Association for Research in Vision and Ophthalmology (ARVO) Statement for the Use of Animals in Ophthalmic and Vision Research (2010/63/EU). One eye only per animal was included, according to ARVO guidelines.

All 8 eyes underwent 23 g pars plana vitrectomy and the vitreous cavity was filled with silicone oil. Three months later, the animals were sacrificed. The eyes were examined histologically for any damage or retinal toxicity by an experienced ocular pathologist (MR).

### Methods: surgical procedure

Prior to surgical procedures, the rabbits were anesthetized by an intramuscular injection of ketamine hydrochloride (30 mg/kg) and xylazine hydrochloride (5 mg/kg). Pupils were dilated using 2.5% phenylephrine/0.5% tropicamide eye drops. Povidone iodine 5% was applied to the eyelids. In addition, topical anesthesia was administered using several drops of lidocaine 0.4%. Two 23ga trocars, inserted in the superotemporal and superior-nasal quadrants (1.0 mm posterior to the corneoscleral limbus were used to insert a lighted infusion cannula and a vitrector (Millennium, Bausch & Lomb, Bridgewater, NJ). Under constant infusion of a balanced saline solution, as much vitreous was removed as possible, while avoiding damage to the lens. After fluid-air exchange, the vitreous cavity was filled with silicone oil. Intraocular pressure was not measured.

Subconjunctival injection of gentamicin and betamethasone depot (Celestone Chronodose, MSD) was administered. Moxifloxacin (Vigamox, Alcon, Fort Worth, TX, USA) drops and tobramycin eye ointment (Alcon, USA) were applied locally.

### Methods: silicone oils used in the experiment

Commercially available, industrial grade dimethylsilicone oils (Xiameter, Dow Corning, Midland, MI, USA) were injected into 6 eyes: 4 received 12,500 cS oil [12] and 2 received 30,000 cS oil [13]. Details of the chemical and physical properties of the silicone oils used are listed in Table 1. Boiling point, optical clarity, and refractive index were the same.

**Table 1:** Chemical and physical properties of the silicone oils evaluated

Properties	Currently used		Tested oils	
Viscosity (cS)	1000	5500	12,500	30,000
Color			Colorless	N/A
Flash point, open cup (°C)	> 326	> 321	> 326	> 326
Flash point, closed cup (°C)	> 100	> 100	> 100	> 100
Hydrophobic	Yes	Yes	Yes	Yes
Pour point (°C)	− 50	− 50	− 50	− 43
Refractive index	1.403	1.403	1.403	1.403
Service temperature, high (°C)	200	200	200	200
Service temperature, low (°C)	−40	− 40	− 40	− 40
Specific gravity @ 25°C	0.971	0.975	0.975	0.971
Surface tension (mN/M)	21.2	21.4	21.5	21.5
Thermal conductivity (W/mK)	0.159	0.159	0.159	N/A
Volatile content			N/A	N/A

The experimental oils were sterilized in an autoclave prior to injection. Two eyes were injected with 5500 cS medical grade, silicone oil (ArcadOphtha, Toulouse, France) currently used clinically.

### Methods: histological examination

Three months after vitrectomy surgery with silicone oil tamponade, the animals were sacrificed. The eyes were enucleated and immediately fixed in 4% paraformaldehyde. After fixation, the globes were vertically sectioned, processed and embedded in paraffin. The hematoxylin and eosin stained sections were evaluated by an experienced ocular pathologist (MR) using light microscopy. The pathologist was blinded to the type of oil inserted into each eye.

### Methods: feasibility of using current vitrectomy system

To examine whether high viscosity silicone oils are readily injectable with the vitrectomy system in use in our hospital (Constellation Vision Systems, Alcon, Fort Worth, TX, USA), we measured the time needed to inject 5 cc of each type of silicone oil, which was taken as an average human vitreous volume, using the VFC Pac and the Constellation Vision System (Alcon, Ft. Worth, TX, USA). The injection times for 1300, 12,500, and 30,000 cS oils, using the 20ga, 23ga, and 25ga injection cannulas supplied with the VFC Pac were measured.

## Results

### Histological examination

Three months after injection with silicone oil, retinal histological examination was performed, including 2 eyes with 5500 cS, 4 eyes with 12,500 cS and 2 eyes with 30,000 cS viscosities.

**Fig. 1:**
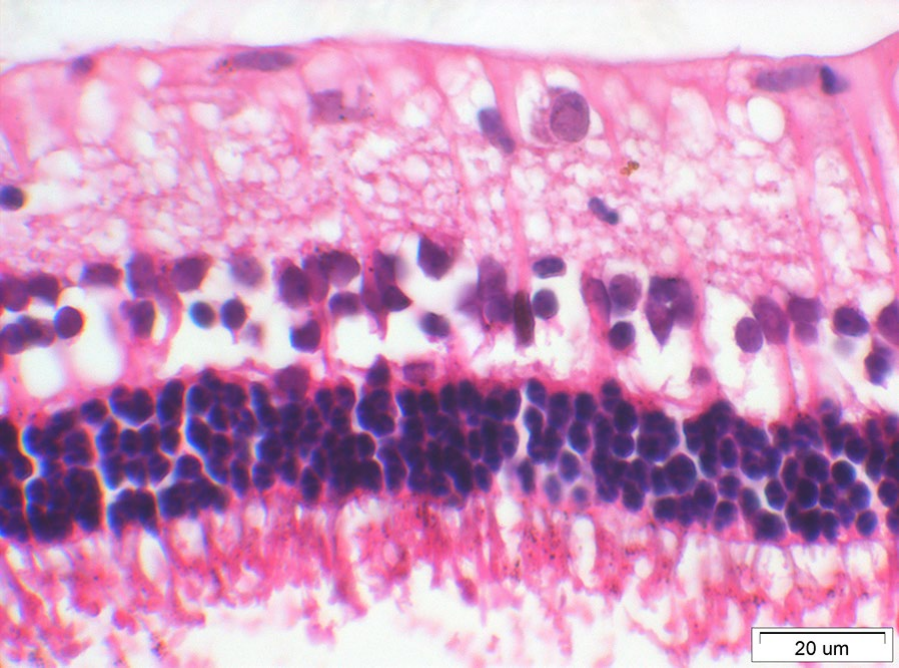
HE × 400, retina of an eye injected with 5500 cS silicone oil

The anterior segments were normal in the two eyes injected with 5500 cS silicone oil. The retina was normal except for artificial changes and inflammatory cells seen in the vitreous cavity of one eye (Fig. 1).

**Fig. 2:**
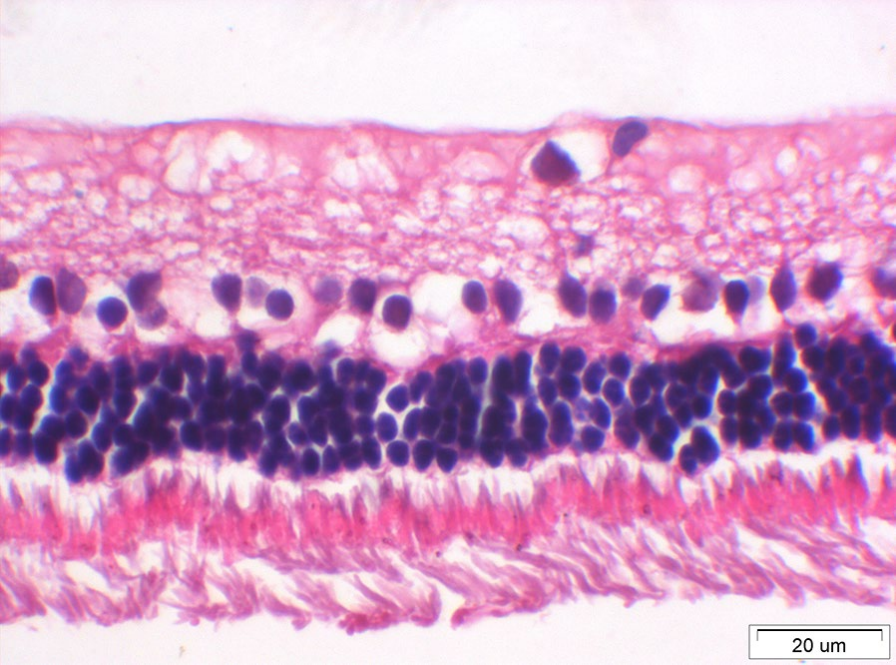
HE × 400, retina of an eye injected with 12,500 cS silicone oil

The anterior segment was normal in all four eyes injected with 12,500 cS silicone oil. The retina was unremarkable except for artificial changes in three eyes. A local area with atrophic changes in the anterior retina was seen in one eye (Fig. 2).

**Fig. 3:**
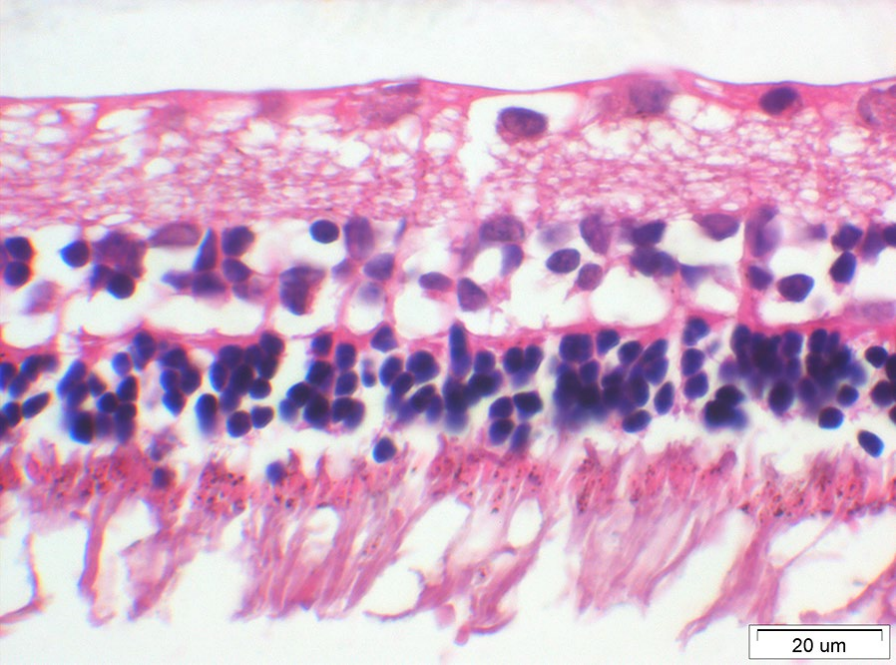
HE × 400, retina of an eye injected with 30,000 cS silicone oil

The anterior segment was normal in the two eyes injected with 30,000 cS silicone oil. The retina was normal, except for a local equatorial area with atrophic changes and some artificial changes showing thin nuclear layers (Fig. 3).

### Injection times

**Fig. 4:**
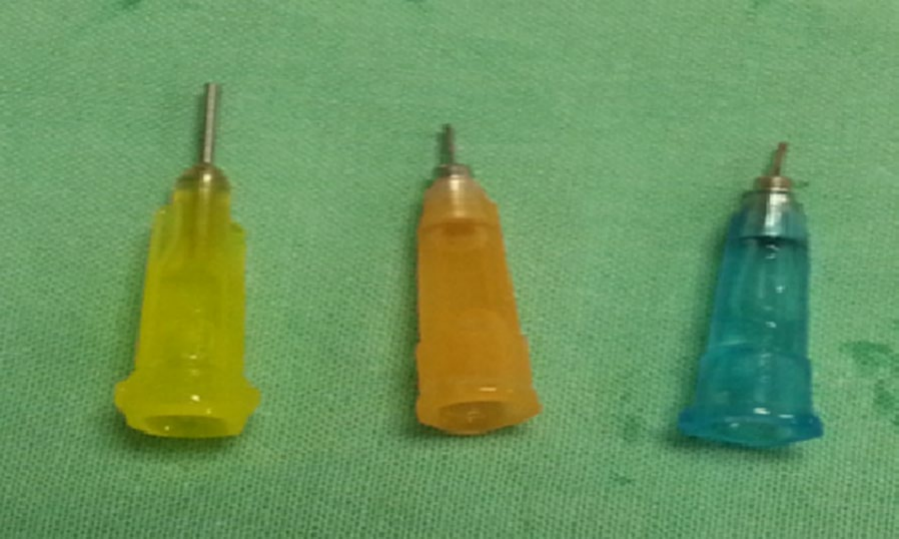
Alcon VFC Pac silicone oil injection cannulas: left—20ga (yellow), middle—23ga (orange), right—25ga (blue)

The injection times for the 3 different viscosities, using the 3 cannula gauges are shown in (Table 2). Unexpectedly, injection times with the 20ga cannula were longer than they were with the 23ga cannula (Fig. 4).

**Table 2:** Injection Times (in seconds) to inject 5 cc for 3 oil viscosities, using the 3 cannula gauges supplied in the VFC PAC

Parameters (cS)	VFC PAC cannula gauge		
	20ga	23ga	25ga
1300	30	13	25
12,500	240	90	180
30,000	540	180	450

## Discussion

This experimental study evaluated the toxicity and safety of using experimental, high viscosity, silicone oils, as well as the feasibility of their use with a currently available vitrectomy system.

Scott et al. compared commercially available 1000 and 5500 cS silicone oils and did not find any clinically significant differences in emulsification [8]. Several in vitro studies demonstrated that higher viscosity silicone oils tend to emulsify less [9–11]. These findings led to several investigations of high viscosity silicone oils [14–18]. However, the silicone oils used in these studies were obtained by adding low concentrations (5–10%) of long chain silicone molecules to regular silicone oil, and not by using uniformly high viscosity oils, as in the current study. We could not find studies in the literature using pure, unadulterated silicone oil as was used in the current study.

This study used an animal model to demonstrate that 3 months of tamponade with high viscosity 12,500 and 30,000 cS silicone oils was well-tolerated and did not cause excess toxicity compared to medically available 5500 cS silicone oil. This agrees with theoretical expectations, as the chemical and physical properties of these oils are identical. The different viscosities are related only to varying molecular chain lengths.

We also demonstrated that silicone oils with viscosities up to 30,000 cS were readily injectable with a currently available vitrectomy system and that the 23ga cannula required the shortest injection duration. We believe this is because the longer 20ga cannula in the VFC Pac caused greater resistance than the much shorter, 23ga cannula. In addition, we found that with some practice, they are simple to use and do not complicate or significantly prolong surgery. As we expect that these high viscosity oils may be particularly useful in refractory cases of failed and redetached retinas after multiple previous surgeries, adding a few minutes to these last resort surgeries would be a minor inconvenience.

## Conclusions

This pilot study suggests that the high viscosity silicone oils tested may be as safe and nontoxic as currently available oils. We hypothesize that high viscosity oils may be less prone to emulsification complications, and may provide effective tamponade in patients who experienced detachment after receiving lower viscosity silicone oils. Therefore, we are continuing to study the properties and use of these oils in retinal detachment.
